# Analysis of *ALK* gene in 133 patients with breast cancer revealed polysomy of chromosome 2 and no *ALK* amplification

**DOI:** 10.1186/s40064-015-1235-9

**Published:** 2015-08-21

**Authors:** Matthew G. Hanna, Vesna Najfeld, Hanna Y. Irie, Joseph Tripodi, Anupma Nayak

**Affiliations:** Department of Pathology and Laboratory Medicine, The Mount Sinai Hospital and Icahn School of Medicine at Mount Sinai, 1 Gustave L. Levy Place, Box 1194, New York, NY 10029 USA; Department of Oncological Sciences, The Mount Sinai Hospital and Icahn School of Medicine at Mount Sinai, New York, NY USA

**Keywords:** *ALK*, Breast cancer, Polysomy, Inflammatory, FISH, TMA

## Abstract

*ALK* has emerged as a novel tumorigenic factor in several epithelial human cancers. Crizotinib, an *ALK* tyrosine kinase inhibitor, is currently approved to treat lung cancer patients exhibiting *ALK* gene rearrangements. Our goal was to determine the incidence of *ALK* aberrations in relation to different breast cancer types. Tissue micro-arrays were constructed of ER+/PR±/HER2− (n = 37), ER−/PR−/HER2+ (n = 15), ER−/PR−/HER2− (n = 61) and ER+/PR+/HER2+ (n = 20) breast cancers; including 13 inflammatory breast carcinomas. FISH was performed using *ALK* break-apart and chromosome 2 centromere enumeration probes (CEP2). Neither *ALK* rearrangements nor amplification were identified in the 133 breast cancer cases evaluated. However, copy number gains (CNG) of *ALK* were identified in 82 of 133 patients (62 %). The CEP2 analysis revealed polysomy of chromosome 2 in all HCNG and LCNG cases, indicating the CNG of *ALK* are due to polysomy of chromosome 2, rather than true amplification of *ALK*. To conclude, we observed CNG of *ALK* secondary to chromosome 2 polysomy in a significant percentage of breast cancer cases, a phenomenon similar to polysomy 17. This study is one of the largest studies to have investigated *ALK* aberrations in breast cancer and the only study to include all subtypes.

## Background

The anaplastic lymphoma kinase (*ALK*), a gene located on the short arm of chromosome 2 (2p23), encodes a transmembrane tyrosine kinase receptor capable of activating downstream *STAT3, AKT/PI3K* and *RAS/ERK* signaling pathways responsible for cell proliferation, migration and survival (Porter and Vaillancourt 1998; Shaw et al. 2011). Alterations in the *ALK* gene are known to play role in genesis and biology of many tumors including anaplastic large cell lymphoma, neuroblastoma, inflammatory myofibroblastic tumors, diffuse large B cell lymphoma, renal carcinoma, serous carcinoma of the ovary, esophageal squamous cell carcinoma, colon carcinoma and more recently non-small cell lung carcinoma. These alterations embrace various rearrangements/translocations, somatic mutations, copy number gains or amplifications (Hallberg and Palmer 2013; Salido et al. 2011; Chen et al. 2008; Lamant et al. 2000; Miyake et al. 2002; Cessna et al. 2002; Passoni et al. 2009; Dirks et al. 2002; Soda et al. 2007; Li et al. 2013). The discovery of the *EML4*-*ALK* fusion gene in a subset of non-small cell lung carcinoma (3–7 %) has steered the development of a therapeutic drug crizotinib (a tyrosine kinase inhibitor that inhibits the activity of the ALK fusion proteins, cMET, ROS1, and RON) that is FDA approved for treating lung cancer patients exhibiting *ALK* gene rearrangements (Zou et al. 2007; Kwak et al. 2010; Sahu et al. 2013).

A few recent studies have investigated the role of *ALK* aberrations in breast cancer, particularly in the context of inflammatory breast cancer and triple negative breast cancers, however, the results have been conflicting (Fukuyoshi et al. 2008; Lin et al. 2009; Robertson et al. 2013; Krishnamurthy et al. 2013; Grob et al. 2012). Inflammatory breast cancer and triple negative breast cancers have the worst prognosis amongst all the subtypes of breast cancer (Engstrøm et al. 2013; Robertson et al. 2010). Inflammatory breast carcinoma is a rare but aggressive type of locally advanced breast cancer characterized by a clinical diagnosis of rapid onset erythema and/or edema of the breast skin (peau d’orange appearance) secondary to blockade of dermal lymphatics by tumor emboli. Most inflammatory breast cancers are hormone receptor (estrogen and progesterone receptors) negative and human epidermal growth factor receptor (HER2) positive, and are treated by multidisciplinary approaches involving systemic chemotherapy, surgery, and radiation. Despite multimodality therapy, overall survival is only 35–40 % at 5-years as compared to 80 % for non-inflammatory breast cancers (Robertson et al. 2010). Triple negative breast cancer is defined by lack of expression of estrogen receptor (ER), progesterone receptor (PR) and human epidermal growth factor receptor (HER2). Due to lack of hormone receptors and HER2 overexpression, there is no effective targeted therapy so far for this particular subset of patients. Therefore, identification of aberrations in genes such as *ALK* will be clinically useful for breast cancers, given the availability of various *ALK* inhibitors.

The purpose of this study was to determine the incidence and frequency of *ALK* amplification and rearrangements in different breast cancer subtypes including triple negative and inflammatory breast cancers.

## Methods

### Case selection

A database search was performed under the approval of our Institutional Review Board for cases meeting clinico-pathologic diagnostic criteria for inflammatory breast carcinoma (between 2001 and 2013), triple negative breast carcinoma (between 2005 and 2013), and other select breast tumor subtypes including ER+, Her2+, and triple+ cancers (between 2012 and 2013). Slides and formalin fixed paraffin embedded (FFPE) tissue blocks of breast tumors were retrieved from pathology archives. Hematoxylin and eosin slides of tumor tissue from resection specimens were reviewed and biomarker profile was recorded from pathology records. American Society of Clinical Oncology (ASCO)/College of American Pathologists (CAP) 2010 Guidelines were used to score the estrogen, progesterone and HER2 protein expression.

### Tissue microarray

**Fig. 1:**
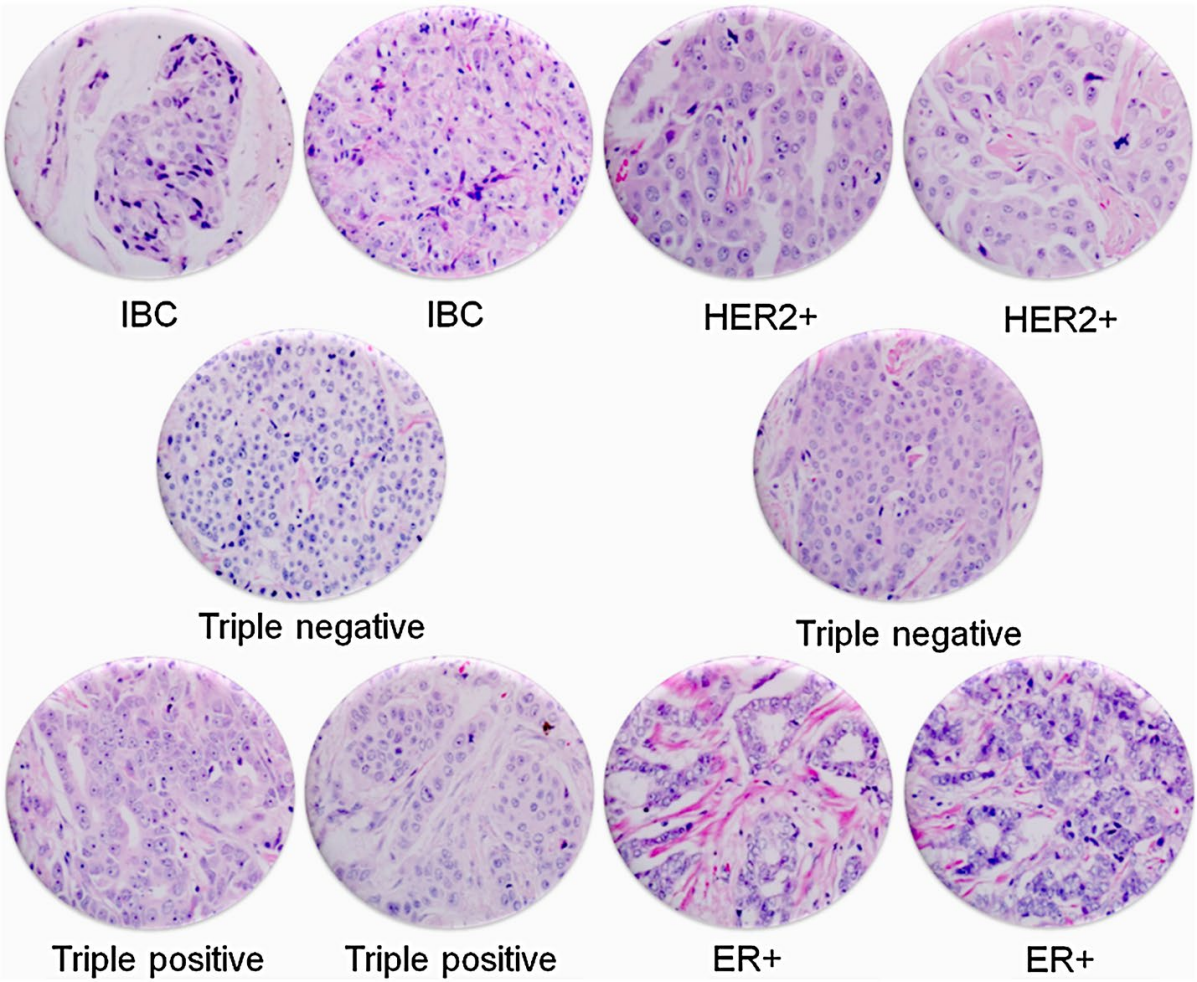
Representative tissue microarray. *IBC* inflammatory breast cancer, HER2 positive [ER−/PR−/HER2+], triple negative [ER−/PR−/HER2−]; triple positive [ER+/PR+/HER2+], ER positive [ER+/PR±/HER2−]

Tissue microarrays (TMA) were constructed with a Chemicon International ATA-100 using two representative 2-mm cores from invasive breast cancers stratified as follows: estrogen receptor positive [ER+/PR±/HER2−], triple positive [ER+/PR+/HER2+], triple negative [ER−/PR−/HER2−], HER2 only positive [ER−/PR−/HER2+] (Fig. 1).

### Fluorescence in-situ hybridization (FISH) for *ALK* gene rearrangement

FISH studies were performed using *ALK* break apart probe [3′-spectrum orange, 5′-spectrum green] (Empire Genomics, Buffalo, NY, USA) and chromosome 2 centromere enumeration probe (CEP2) [spectrum aqua] (Abbott Molecular, Des Plaines, IL, USA) in all cases. Three micron thick tissue sections were de-paraffinized and pretreated using a Dako (Dako, Carpinteria, CA, USA) de-paraffinization and pretreatment technique followed by hybridization with a probe mixture (*ALK* and CEP2) of 10 µl at 45 °C on Hybrite (Abbott Molecular). After hybridization, the slides were washed and counterstained with DAPI.

**Fig. 2:**
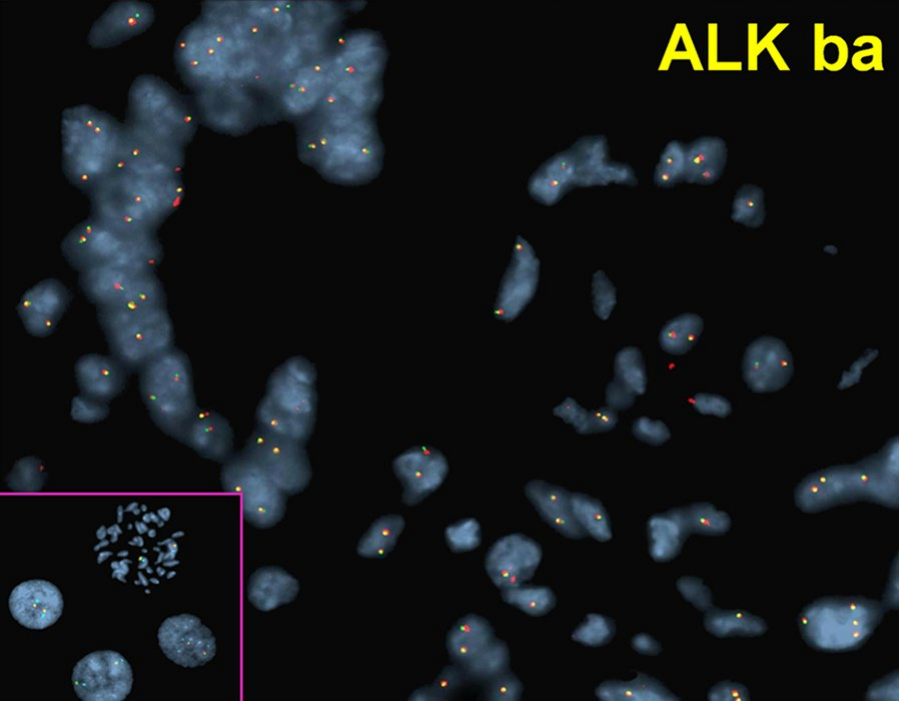
FISH of normal breast terminal duct lobule showing normal disomy for ALK [3′-spectrum orange, 5′-spectrum green] signals (*yellow*); a normal pattern in non-tumor breast cells. *Inset* CEP2 probe [spectrum aqua] performed on metaphase and control cells with normal 2 signal pattern

*ALK* probe validation was carried out on FFPE sections from non-tumor breast tissue and tonsil tissue. A total of 5000 cells were evaluated and scored along with the CEP2 [spectrum aqua]. Ninety-seven percent of non-tumor cells had 2 aqua and 2 *ALK* fusion signals (yellow), the other 3 % showed variations of normal signal patterns (Fig. 2). The reference range was determined to be <6 % of cells, whereas any abnormal signal pattern in ≥6 % of cells was considered abnormal.

### FISH analysis

At least 100 tumor cells per case were evaluated in 133 patients with breast cancer. Low copy number gain (LCNG) was defined as 3–6 *ALK* signals, and high copy number gain (HCNG) with >6 *ALK* signals in ≥6 % of tumor cells. Amplification was defined as >6 copies of *ALK* signals with no more than 2 centromere enumeration probe 2 (CEP2) aqua signals in >15 % of cells. Positive rearrangement was defined if 1 set of orange and green signals were >2 signals apart in >15 % of tumor cells, or when a single orange signal with a separate fusion (shown as one yellow signal) was seen in >15 % tumor cells. Two different observers performed evaluations to reduce inter-observer variability.

### *ALK* immunohistochemical staining

Immunohistochemistry for ALK protein was performed only in IBC cases, using standard laboratory protocols in Dako Autostainer Link 48. The de-paraffinized tissue microarray of the IBC cases (n = 13) was pretreated with heat-induced epitope retrieval: the slide was pre-heated to a temperature of 65 °C; the epitope retrieval temperature was 97 °C for 20 min followed by cooling down to 65 °C. The slide was then washed into diluted EnVision FLEX Wash Buffer at 22 °C for 3 min and then incubated for 20 min with FLEX mouse monoclonal anti-human CD246 ALK antibody (clone ALK1, Dako, Carpinteria, CA, USA) and FLEX+, mouse, Linker. Positive and negative controls were performed simultaneously. Positive staining was defined as brown cytoplasmic positivity in the tumor cells.

## Results

The distribution of our 133 study cases is as follows: ER+/PR±/HER2− (n = 37), ER−/PR−/HER2+ (n = 15), ER−/PR−/HER2− (n = 61), ER+/PR+/HER2+ (n = 20). Of these 133 cases, 13 had a clinico-pathologic diagnosis of inflammatory breast cancer and include 3 ER−/PR−/HER2− (24 %), 5 ER−/PR−/HER2+ (38 %), and 5 ER+/PR±/HER2− (38 %) cases.

None of the 133 cases showed *ALK* rearrangement or amplification. However, copy number gains of *ALK* were identified in 82 of 133 patients (62 %). Of these 82 cases, four (5 %) demonstrated HCNGs of ALK (range 6–13 % of tumor cells) and include 1 ER−/PR−/HER2−, 2 ER+/PR−/HER2− and 1 HER2 only positive (ER−/PR−/HER2+) case. The remaining 78 cases (95 %) demonstrated low copy number gains and include 12 (80 %) ER−/PR−/HER2+ (range 10–68 % of tumor cells), 39 (64 %) ER−/PR−/HER2− (range 6–68 % of tumor cells), 21 (57 %) ER+/PR±/HER2− (range 6–69 % of tumor cells), and 6 (30 %) ER+/PR+/HER2+ cases (range 6–91 % of tumor cells) (Table 1).

**Table 1:** Signal pattern [n (cell range %)] of *ALK*

	Split pattern (1O1G1F)	Single orange (1O1F)	LCNG	HCNG
ER−/PR−/HER2− (n = 61)	1 (2 %)	12 (1–3 %)	39 (6–68 %)	1 (13 %)
ER−/PR−/HER2+ (n = 15)	0 (0 %)	3 (1–3 %)	12 (10–68 %)	1 (8 %)
ER+/PR±/HER2− (n = 37)	3 (2–3 %)	7 (1–12 %)	21 (6–69 %)	2 (6–11 %)
ER+/PR+/HER2+ (n = 20)	0 (0 %)	1 (1 %)	6 (6–91 %)	0 (0 %)
Inflammatory breast carcinoma (n = 13)	3 (2–3 %)	3 (5–12 %)	10 (9–69 %)	3 (6–13 %)

*1O* one orange signal (3′), *1G* one green signal (5′), *1F* one fusion signal (yellow), *LCNG* low copy number gains, *HCNG* high copy number gains

Since the previous studies on *ALK* are primarily focused on inflammatory breast carcinoma, we analyzed the results of our 13 inflammatory breast carcinoma cases separately. All 13 cases showed low or HCNGs of *ALK* without any rearrangement or amplification. Interestingly 3 of the 4 cases in HCNG cohort were inflammatory breast carcinomas (1 ER−/PR−/HER2− and 2 ER+/PR−/HER2−; range 6–13 % of tumor cells). The remaining ten cases demonstrated low copy number gains (range 9–69 % of tumor cells).

**Fig. 3:**
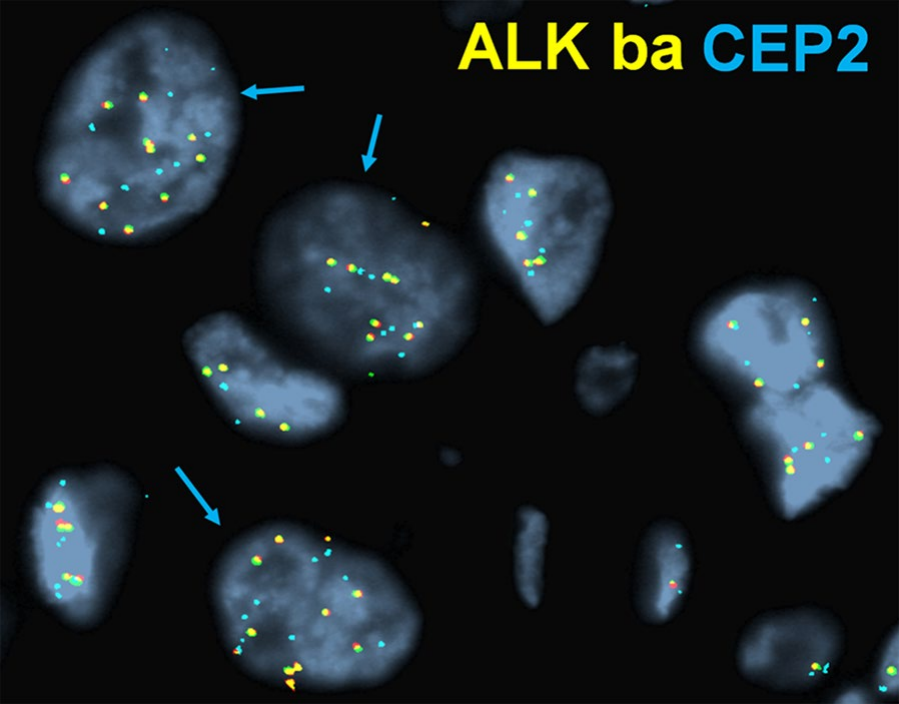
High copy number gain and chromosome 2 polysomy. A case of inflammatory breast cancer revealing >6 signals/cell of ALK break-apart probes (HCNG) in representative tumor cells (*closed-head arrows*). Note, the same tumor cells show >2 CEP2 (aqua) signals, indicating polysomy for chromosome 2

**Fig. 4:**
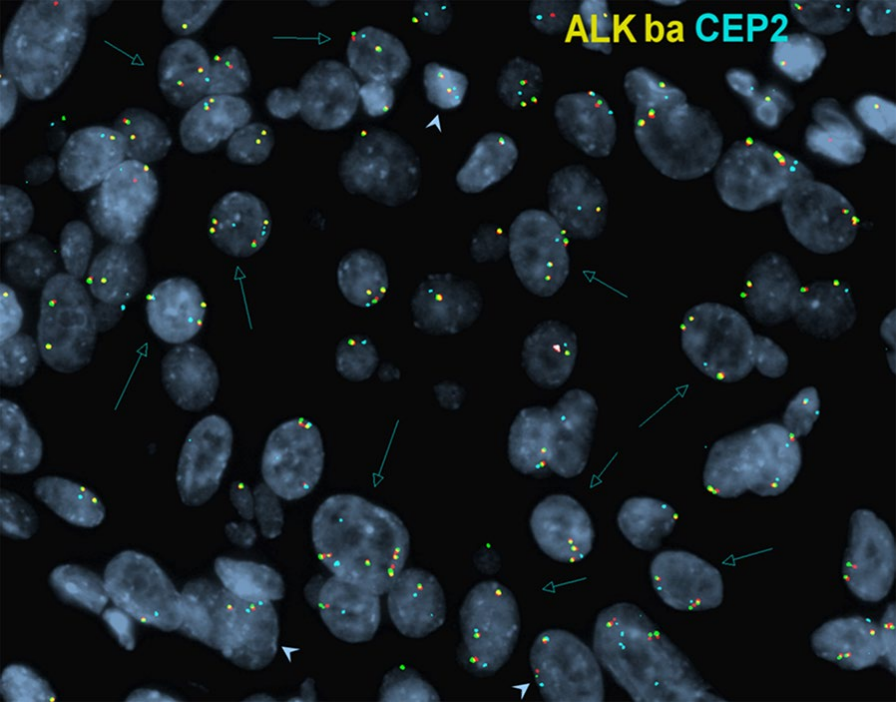
Low copy number gain. A case of HER2+ breast cancer is shown revealing 3–6 ALK signals of ALK break-apart probes in representative tumor cells (*open-head arrows*). Normal signal pattern are seen in cells (*arrowheads*), as 2 ALK (*yellow*) signals with 2 CEP2 (aqua) signals

The CEP2 analysis in HCNG cases revealed polysomy of chromosome 2 in all 4 (100 %) cases with 3–16 CEP2 signals noted in 51–64 % of tumor cells (Fig. 3). The CEP2 analysis in the remaining cases with low copy number gains also showed chromosome 2 polysomy (3–8 signals/cell, range 6–87 % of tumor cells) (Fig. 4).

Immunohistochemical staining for ALK protein expression was only performed in the IBC cases (n = 13) and none of the cases expressed ALK protein.

## Discussion

Our study demonstrated that *ALK* gene was neither rearranged nor amplified in any of the 133-breast cancer cases evaluated by FISH. However, a significant percentage of cases (82/133; 62 %) revealed low copy number gains of *ALK* irrespective of the subtype. HCNGs of *ALK* were limited to a subset of inflammatory breast cancer (3, 23 %), and a single ER−/PR−/HER2+ (1, 7 %) case. All other tumor subtypes showed only low copy number gains. The two subtypes with the most copy number alterations were the inflammatory breast cancer (100 %) and ER−/PR−/HER2+ cases (87 %). FISH testing for CEP2 probe demonstrated that increased copy number gains of *ALK* are due to polysomy of chromosome 2 rather than true amplification of *ALK* gene.

There are very few studies exploring *ALK* gene aberrations in breast cancer, and the results yielded have been conflicting. In 2008, Fukuyoshi et al. used *EML4*-*ALK* transcripts to detect the incidence of *EML4*-*ALK* rearrangements in lung, colon, and breast cancer. Their study did not detect any *EML4*-*ALK* rearrangements in 90 breast cancer cases included in the study (Fukuyoshi et al. 2008). Conversely Lin et al. (2009) identified *EML4*-*ALK* rearrangements in 5 of 209 (2.4 %) breast cancers (subtype unspecified) using RT-PCR exon array genomic sequencing and confirmatory FISH testing. Robertson et al. exclusively studied inflammatory breast cancers using Reverse Phase Protein Microarray analysis, a pathway activation mapping technique, and reported activation of multiple members of the receptor tyrosine kinase *ALK* signaling network (*JAK1/STAT3, AKT, mTOR, PDK*-*1* and *AMP kinase β*) in inflammatory breast cancer cell lines, at levels similar to those in *ALK* rearranged non-small cell lung cancers. Authors extended their study to clinical tumor samples of inflammatory breast cancer and detected *ALK* copy number gains and gene amplification in 20/25 (80 %) patient tumor samples. They also identified one case with an *EML4*-*ALK* translocation. Furthermore, these investigators successfully demonstrated that even the submicromolar concentrations of crizotinib induced significant tumor shrinkage in the pre-clinical xenograft models of inflammatory breast cancer. Currently a phase I clinical trial is enrolling patients with inflammatory breast carcinoma for treatment with crizotinib (Robertson et al. 2013). In contrast, Krishnamurthy et al. (2013) did not detect *EML4*-*ALK* gene rearrangement or *ALK* protein expression in the inflammatory breast cancer cases. There is only one study published to date addressing triple negative breast cancers and did not report *EML4*-*ALK* rearrangement in 65 cases tested using FISH (Grob et al. 2012). Although the results of aforementioned studies are inconsistent, they collectively suggest lower incidence of *EML4*-*ALK* rearrangements in breast cancer as compared to non-small cell lung cancer.

Our study is one of the largest clinical studies to have investigated *ALK* gene rearrangements and amplification in breast cancer and the only study to include all subtypes. This study highlights that copy number gain of *ALK* gene is the result of polysomy of chromosome 2 and reflects a common phenomenon in all breast cancer subtypes, specifically in inflammatory and ER−/PR−/HER2+ breast cancers.

The functional significance of *ALK* copy number gains or polysomy2 in breast carcinogenesis is not clear. Recent study by Kim et al. demonstrated that *ALK* CNG was observed in 17/36 (47.2 %) IBC cases and was associated with worse overall survival when compared to the patients without *ALK* CNG by univariate analysis (24.9 vs 38.1 months; p = 0.033). The recurrence free survival after mastectomy was also shorter in the patients with *ALK* CNG (12.7 vs 43.4 months; p = 0.016) (Kim et al. 2015). Studies of *ALK* in solid tumors other than breast have shown similar correlation with worse prognosis. Jia et al. (2014) reported that increased *ALK* copy number gains in hepatocellular carcinoma are associated with a decreased 3-year overall survival (18.2 vs 63.6 %; p = 0.021) and 3-year progression free survival (18.2 vs 46.9 %; p = 0.019) as compared to those without *ALK* copy number alterations. In lung cancer, copy number gain of *ALK* is a more frequent phenomenon than *ALK* rearrangement, and is reported in up to 63 % of cases (Salido et al. 2011). This has persuaded investigators to study if tyrosine kinase inhibitors would be effective even in patients with *ALK* copy number gains without rearrangement; however, the results have been conflicting. A recent study tested crizotinib sensitivity in nine lung cancer cell lines and three patients with NSCLC exhibiting *ALK* CNG, and concluded that HCNG without *ALK* rearrangement has predictive value for chemo sensitivity to crizotinib (Kalai et al. 2012). On the contrary; CNG of *ALK* has been implicated as one of the mechanisms for developing resistance to tyrosine kinase inhibitors. Pietrantonio et al. (2014) have reported that gain of *ALK* gene copy number may predict lack of benefit from anti-EGFR treatment in patients with advanced colorectal cancer and RAS-RAF-PI3KCA wild-type status. It would be of interest to study whether *ALK* tyrosine kinase inhibitors would be effective in breast cancers with *ALK* copy number gains.

In our study, none of the 13 inflammatory breast cancer cases (including 3 cases with high CNG) exhibited ALK protein overexpression by immunohistochemistry. This is in contrast to the study by Kim et al. (2015) where ALK protein expression was demonstrated in 15/27 inflammatory breast cancer cases. However, no significant correlation was observed between CNG and ALK protein expression. Nine of 14 IBC cases without CNG revealed ALK protein expression compared to 6 of 13 cases with CNG (p = 0.767).

## Conclusion

Neither *ALK* gene rearrangements nor amplification were identified in the 133 breast cancer cases evaluated in our study. We observed extra copy numbers of *ALK* as a result of chromosome 2 polysomy in 62 % of breast cancer cases. This suggests polysomy of chromosome 2 is common in breast cancer regardless of the subtype, a phenomenon analogous to polysomy 17. The clinical and functional significance of chromosome 2 polysomy and extra copy numbers of *ALK* in breast cancer is yet to be elucidated. Larger studies with survival data are needed in the context of breast cancer to draw such conclusion.
